# Thermal, Crystallization, and Toughness Behavior of Polyamide 4/Long-Chain Hyperbranched Polymer Blends

**DOI:** 10.3390/polym17030318

**Published:** 2025-01-24

**Authors:** Linyi Shui, Xianxin Guo, Jinrong Li, Zimeng Li, Qinghua Zhao, Guohua Chen, Xiaomin Zhao

**Affiliations:** College of Materials Science and Engineering, Huaqiao University, Xiamen 361021, China; 18208383284@163.com (L.S.); g181208@163.com (X.G.); 18172673361@163.com (J.L.); 19859293281@163.com (Z.L.); qhzhao@hqu.edu.cn (Q.Z.); hdcgh@hqu.edu.cn (G.C.)

**Keywords:** polybutyractam, long-chain hyperbranched polyester, toughening, thermal stability

## Abstract

Long-chain hyperbranched polyesters (LHBPx, x = 1, 2, 3) with varying lengths of branched chains were synthesized through a thiol-ene click reaction. Subsequently, LHBPx was incorporated into PA4 via the solution method to prepare a LHBPx/PA4 polymer blend, aiming to address the limitations of PA4, such as its narrow thermal processing window (△T = T_d5_−T_m_) and high brittleness. The results demonstrated that the addition of LHBPx enhanced the △T of PA4 from 1.6 °C to 14.5 °C (LHBP_3_/PA4), increasing the rheological properties of LHBPx/PA4 polymer blends, thereby improving its thermal processability. Compared with PA4, the elongation at the break of the LHBP_3_/PA4 polymer blend was increased by 20.4%, and the brittle fracture was changed into a ductile fracture. The crystallinity of PA4 was greatly decreased, from 54.41% to 37.42%, owing to the incorporation of LHBPx, whereas T_m_ of PA4 had almost no change. It was explained that LHBPx hindered the crystal growth stage, whereas it promoted the nucleation stage of PA4, resulting in no significant change in crystal type. Moreover, the longer the branched chain of LHBPx was, the more pronounced the improvement in the thermal processability and toughness of PA4 became. Above all, this work was meaningful for the potential application of PA4 in industrial plastics.

## 1. Introduction

PA4 (Polyamide 4) can be completely degraded naturally within a few months, making it a renewable raw material with environmentally friendly and other characteristics, but it also has heat resistance, impact resistance, and gas barrier characteristics [1] as good as the market PA6 and PA66 [2], which makes it useful for biological tissue engineering [3], packaging engineering [4], and as an engineering plastic [1] in the construction, automotive, and other fields, with very good advantages in those applications. However, the dense distribution of amide groups along the PA4 molecular chain results in strong intermolecular hydrogen bonding, leading to challenges such as a narrow processing temperature range and brittleness [5,6]. Therefore, enhancing thermal processability and toughness represents a crucial focus in PA4 research.

Some researchers have used various techniques, such as copolymerization [7,8,9,10,11] and splicing or end sealing [12], to modify the molecular chain structure of PA4. However, these intrinsic modifications may compromise PA4′s biodegradability and have some disadvantages such as a complex synthesis process and wide molecular weight distribution of products. Additionally, simpler blending methods like solution-blending film layering [13,14], solution electrostatic spinning [15,16], or melt extrusion [17] have also been employed by researchers for non-intrinsic modification of PA4. Nevertheless, doping modifications frequently encounter compatibility issues, which further result in phase separation within the polymer blend, adversely affecting their mechanical properties [18].

Long-chain hyperbranched polymer (LCHBP) combines the advantageous features of hyperbranched polymers (HBPs) [19,20] and linear polymers, having benefits such as reduced hydrodynamic volume, lower viscosity, and enhanced inter-molecular entanglement [21,22]. Incorporating LCHBP into linear polymer matrices can effectively enhance both toughness and processability [23,24]. Yang et al. [22] found that linear triblock copolymer macromolecules have a distinct long-range ordered structure, whereas the macromolecular monomers generate LHBP without a distinct long-range ordered structure. Li et al. [25] studied the rheological properties and aggregation morphology of LHBP. They found that an increase in the degree of aggregation of LHBP is not conducive to intermolecular chain entanglement and that LHBP in different solvents may collapse or aggregate. Konkel et al. [24] obtained LCHBP by linking a large number of fatty acid long chains to the ends of hyperbranched polyesters and extruded LCHBP with PLA/PPC melt blending, and it was found that the addition of LCHBP improved the compatibility between PLA and PPC, and the addition of 2% of LCHBP increased the impact strength by 61% and the elongation at the break by 367%. LHBP combines the advantages of both hyperbranched polymer and linear polymers, retaining the benefits of small hydrodynamic volume and low viscosity, but also increasing intermolecular chain entanglement [21]. When modified to linear polymers, it effectively inhibits the long-range ordering of polymer microphase separation structure, reduces the crystallinity of polymers, and improves the toughness of polymers. Moreover, LHBPx exhibits remarkable designability, enabling the synthesis of three-dimensional typologies with diverse structures through different synthetic approaches [20,26]. Furthermore, the thiol-ene click [27,28] reaction is widely employed to synthesize HBP due to its high selectivity, rapid kinetics, and mild reaction conditions [29].

In this study, the LHBPx with varying branched chain lengths was synthesized by dienyl monomer (A_2_-X), tetrasulfhydryl monomer (B_4_), and trans-methyl crotonate (TMC). The structures of LHBPx were characterized using ^1^hydrogen-nuclear magnetic resonance (^1^H-NMR). Subsequently, LHBPx was incorporated into PA4 via a solution method to obtain the LHBPx/PA4 polymer blend. The inter-molecular hydrogen bonds of LHBPx/PA4 polymer blend were analyzed by a Fourier transform infrared spectrometer (FTIR). The thermal, rheological, and mechanical properties of the LHBPx/PA4 polymer blend were evaluated. The tensile fracture type was analyzed by scanning electron microscope (SEM). Furthermore, an X-ray diffractometer (XRD) was employed to characterize the crystal structure of the LHBPx/PA4 polymer blend, and non-isothermal kinetic calculations were performed to study its crystallization process.

## 2. Experimental

### 2.1. Materials

Gamma-aminobutyric acid (GABA) (Wanxiang Hongrun Biotechnology Co., Ltd., Chengdu, China); benzoyl chloride (Aladdin Biochemical Technology Co., Ltd., Shanghai, China); formic acid (Titan Scientific Co., Ltd., Shanghai, China); pentaerythritol tetra(3-mercapto propionate) (B_4_), polyethylene glycol 200 diacrylate (A_2_-1), polyethylene glycol 400 diacrylate (A_2_-2), polyethylene glycol 1000 diacrylate (A_2_-3), trans-methyl crotonate (TMC), 2,2-azobis(2-methylpropionitrile) (AIBN), tetrahydrofuran, hexafluoroisopropanol, and potassium tert-butoxide were supplied by Adamas Reagent Co., Ltd. (Shanghai, China); acetone, ethanol, methylbenzene, diethyl ether, and trifluoroethanol were supplied by China National Medicines Co., Ltd. (Beijing, China).

### 2.2. Preparation of LHBPx/PA4 Polymer Blend

#### 2.2.1. Synthesis of PA4

**Scheme 1:**
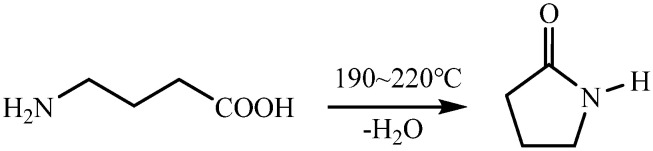
Synthesis equation of BBY.

GABA was slowly heated from 190 °C to 220 °C under nitrogen protection, dehydrated at 220 °C for 2 h, and then purified at 130 °C under reduced pressure to obtain transparent biobutylactam (BBY) (Scheme 1) [30].

The mixture of BBY (4 mL) and tert-butanol methyl (0.283 g) were reacted under vacuum for 2 h at 90 °C, and cooled to 40 °C. Then, a benzoyl chloride (160 μL) initiator was added to the nitrogen atmosphere for an anionic ring-opening reaction for 12 h, dissolved by formic acid (10 mL), and precipitated in acetone (50 mL) to obtain a flocculent precipitate. After washing with deionized water and ethanol, the PA4 product was obtained by vacuum drying at 80 °C for 12 h. The yield of PA4 was 83%, Mη ≈ 21,000.

#### 2.2.2. Synthesis of LHBPx

**Scheme 2:**
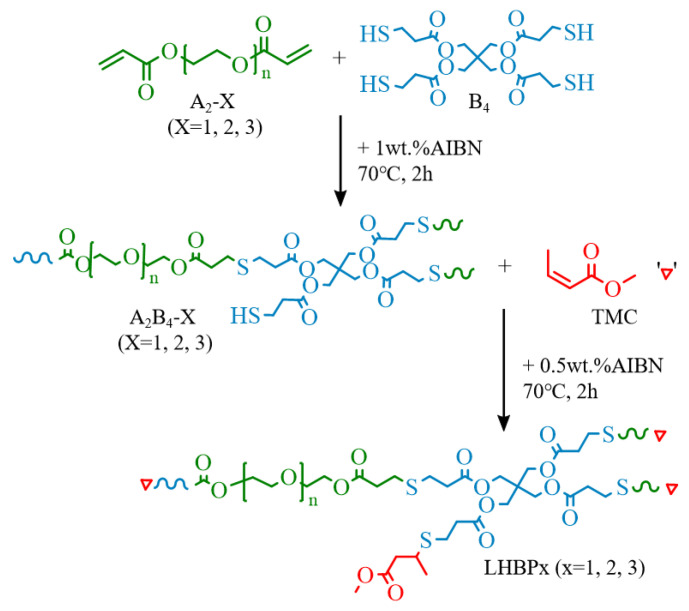
Synthesis route of LHBPx.

The LHBPx was prepared via thiol-ene click reaction. A_2_-X, B_4_, AIBN (0.1 g), and toluene (100 mL) were added to a round-bottom flask and mixed evenly; then, the reaction was carried out at 70 °C under a nitrogen atmosphere for 2 h. The product was added to ether, stirred and mixed for 10 min, the supernatant was poured away, dichloromethane was added to dissolve the product, and repeated three times to remove excess reactant, and then the organic solvent was removed by rotary evaporation. Then, the TMC (20 mL) and AIBN (0.15 g) were added, and continued to react under nitrogen atmosphere at 70 °C for 2 h, adding ether and pouring off the supernatant, dissolving the precipitate with dichloride, and repeated three times to wash away the non-reactants, and then rotating evaporation was used to remove the organic solvent to obtain transparent and viscous LHBPx (Scheme 2). The types and amounts of A_2_-X and B_4_ required for LHBPx synthesis are shown in Table 1. The yield of LHBPx was higher than 94%.

#### 2.2.3. Solution Method to Prepare LHBPx/PA4 Polymer Blends

Each LHBPx/PA4 polymer blend was prepared via a solution-blended process. PA4 and LHBPx were added into the trifluoroethanol solution (solution concentration 10 wt.%) at a 9:1 ratio, stirred at room temperature for 24 h, poured into the silicone mold, and dried at room temperature for 4 h, and then dried overnight in a drying oven at 80 °C. A LHBPx/PA4 polymer blend with a mass fraction of 10 wt.% was obtained. The composite material was placed in a hot-pressing mold, preheated for 1 min at an atmospheric pressure at 230 °C, and then held under 6 MPa pressure for 1 min. After pressure release, the sample was taken out for rapid cooling, and a translucent LHBPx/PA4 polymer blend was obtained for subsequent testing. The compositions of the blends are summarized in Table 2.

### 2.3. Characterization of LHBPx/PA4 Polymer Blends

#### 2.3.1. Hydrogen Nuclear Magnetic Resonance (^1^H-NMR)

The structures of A_2_, B_4_, TMC, and LHBPx were determined by a ^1^H-NMR (AVANCE III, 500 MHz, Bruker Co., Ltd., Billerica, Germany) at concentrations of 25 mg·mL^−1^ in deuterated trifluoroacetic acid (TFA-d) for PA4 dissolution and deuterated chloroform (CDCl_3_) for LHBPx dissolution.

#### 2.3.2. Fourier Transform Infrared Spectrometer (FTIR)

The functional groups of the LHBPx/PA4 polymer blends were characterized by a FTIR (Nicolet IS 50, Thermo Fischer Technology Co., Ltd., Waltham, MA, USA) in attenuated total reflection mode. The test range was 4000~400 cm^−1^ and the scanning times were 32.

#### 2.3.3. Thermogravimetric Analyzer (TGA)

The thermal stability of the LHBPx/PA4 polymer blends was assessed by a TGA (TGA-2, Mettler Toledo Co., Ltd., Zurich, Switzerland). The 6~8 mg samples were placed in aluminum crucibles under N_2_ atmosphere, and the system was heated from 30 °C to 600 °C at a heating rate of 10 °C·min^−1^, with a gas flow rate of 50 mL·min^−1^.

#### 2.3.4. Differential Scanning Calorimeter (DSC)

The thermal properties of the LHBPx/PA4 polymer blends were evaluated by DSC (DSC-200, Netzsch Co., Ltd., Selb, Germany). The 6~8 mg samples were placed in aluminum crucibles under Ar atmosphere, and the system was heated from 30 °C to 270 °C at a heating rate of 10 °C·min^−1^. The non-isothermal crystallization kinetics of the polymer blends were examined via first being heated from 30 to 270 °C at a rate of 10 °C·min^−1^ and then cooled at five different cooling rates (2.5, 5, 10, 20, 40 °C·min^−1^).

#### 2.3.5. Relative Viscosity

The relative viscosity of the LHBPx and LHBPx/PA4 polymer blends was measured by using a rotary viscometer (VISCO, Atago Co., Ltd., Tokyo, Japan) at a concentration of 1 g·dL^−1^ in m-cresol at 25 °C.

#### 2.3.6. Electronic Universal Testing Machine

The mechanical properties of the LHBPx/PA4 polymer blends were evaluated by an electronic universal testing machine (CMT4204, Heng Yu Co., Ltd., Hong Kong, China) at a stretching rate of 50 μm·s^−1^ and a temperature of 25 °C. The samples were cut as follows: the total length was 27 mm and width was 10 mm; the parallel part was 10 mm long and 5 mm wide, and 3~5 groups of each sample were tested.

#### 2.3.7. Scanning Electron Microscope (SEM)

The tensile section morphology of the LHBPx/PA4 polymer blends was characterized by a cold SEM (SU8010, Hitachi Co., Ltd., Tokyo, Japan). The sample was placed on an electron microscope flat plate with conductive tape, sprayed with gold by an ion-sputtering instrument (E-1010, Hitachi Co., Ltd., Japan), and observed with a SEM at an operating voltage of 5 kV.

#### 2.3.8. X-Ray Diffractometer (XRD)

The crystal structure of a LHBPx/PA4 polymer blend was analyzed by an XRD (Smar/SmartLa, Rigaku Co., Ltd., Tokyo, Japan) operating at CuK (λ = 1.542 A). The scanning range was set from 5 to 60 ° at a scanning speed of 10 °·min^−1^.

#### 2.3.9. Gel Permeation Chromatography (GPC) Analysis

The apparent number average (Mn) and weight average molecular weight (Mw) of the samples were characterized by a gel permeation chromatography (GPC) analyzer, model PL-GPC50, from Agilent, Santa Clara, CA, USA. The testing temperature was 25 °C, THF was used as the eluent, the flow rate was 1 mL/min, and linear polystyrene was used as the standard sample.

## 3. Results and Discussion

### 3.1. Chemical Structures and Thermal Properties of LHBPx

#### 3.1.1. Chemical Structures of LHBPx

**Figure 1:**
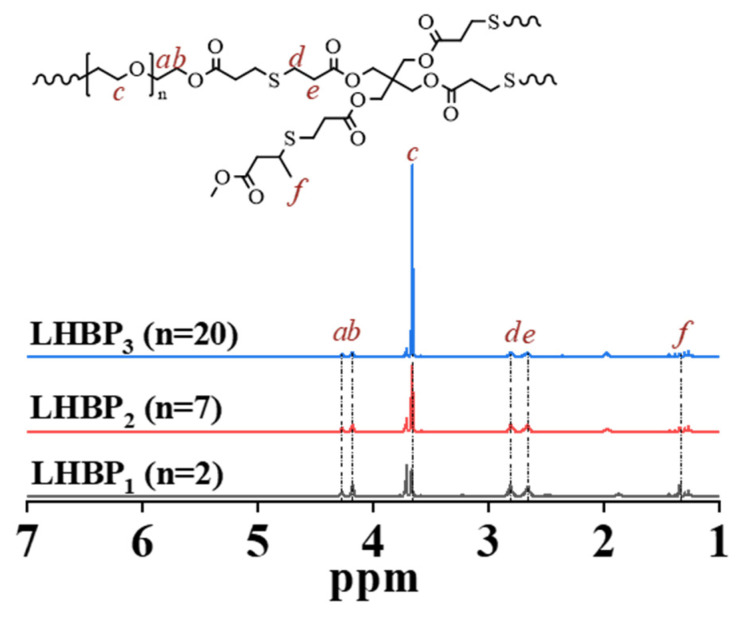
^1^H-NMR spectra of LHBPx.

^1^H-NMR spectroscopy was used to characterize the structure of LHBPx (Figure 1) [31]. The ‘a’ and ‘b’ were the CH_2_ peak of the LHBPx linear unit, ‘c’ was the CH_2_ peak of the ether repeat unit of the LHBPx linear unit, ‘d’ and ‘e’ were the CH_2_ peak of the LHBPx dendritic unit, and ‘f’ was the CH_3_ peak in the LHBPx terminal unit.

The CH_2_=CH_2_ peak (5.2~6.2 ppm) of A_2_-X disappeared (Figure S1), the ‘d’ and ‘e’ peaks of LHBPx appeared, indicating a thiol-ene reaction between A_2_-X and B_4_ had occurred. At 1.65 ppm, the S-H peak (1.5~1.6 ppm) disappeared (Figure S1), and the ‘f’ peak belonging to the terminal reagent TMC appeared, confirming the successful synthesis of LHBPx terminated with TMC. As the branched chain length of A_2_-X increased, A_d_/A_c_ gradually decreased in LHBPx, suggesting an increase in branched chain length and successful synthesis of LHBPx (LHBP_1_, LHBP_2_, and LHBP_3_) with different branched chain lengths.

#### 3.1.2. Gel Permeation Chromatography (GPC) of LHBPx

**Figure 2:**
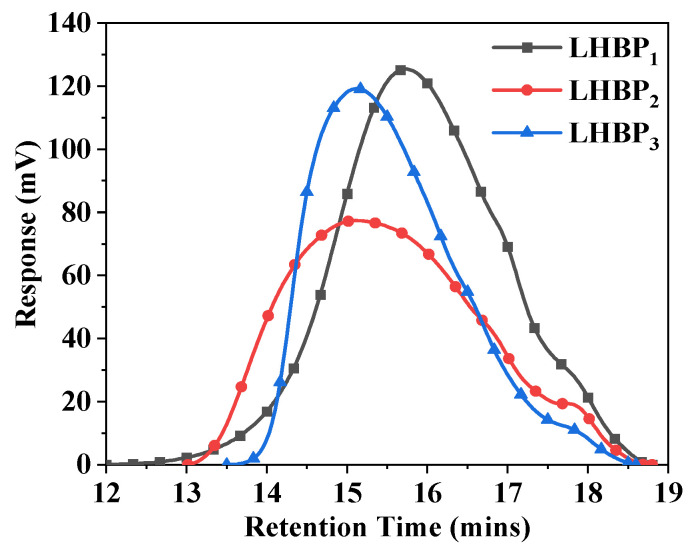
GPC flow curves of LHBPx.

Gel permeation chromatography was used to characterize the molecular weight and molecular weight distribution of LHBPx. Figure 2 shows the GPC efflux time curve of LHBPx. The figure shows that the GPC curves of all hyperbranched polysulfides were single peaks, indicating that the relative molecular weight distribution of the synthesized LHBPx was relatively concentrated, and there was no reactant residue in the reaction system.

The relative molecular weights and molecular weight distributions of the prepared LHBPx were fitted from the GPC outflow curves (the results are shown in Table 3), from which it can be seen that the number average molecular weights of LHBPx increased gradually with the increase in the branched chain lengths. The weight-average molecular weights increased first and then decreased. The polydispersity coefficients of PDI were all greater than 3 and showed a trend of increasing first and then decreasing, which indicated that the molecular weight distributions of LHBPx were wider. The three kinds of weight-average molecular weights of LHBPx with different chain lengths were greater than 15,000.

#### 3.1.3. Thermal Property of LHBPx

**Figure 3:**
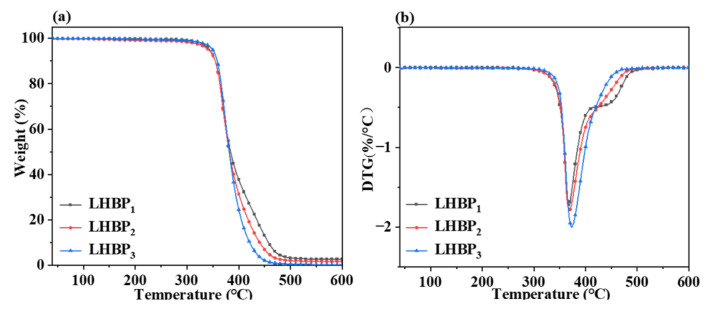
(**a**) TG curve of LHBPx, (**b**) DTG curve of LHBPx.

LHBPx had excellent thermal stability, and the T_d5_ of LHBPx with three different branched-chain lengths was greater than 345 °C. With the increase in the branched-chain length, the T_d5_ of LHBPx gradually increased, and the temperature T_max_ corresponding to the maximum thermal decomposition rate also gradually increased. The peak area in the 360 to 435 °C interval in the DTG profile of Figure 3b increased with the increase in the length of the branched chain, while the peak area in the 435 to 500 °C interval decreased with the increase in the length of the branched chain.

### 3.2. Intermolecular Hydrogen Bonds of LHBPx/PA4 Polymer Blends

**Figure 4:**
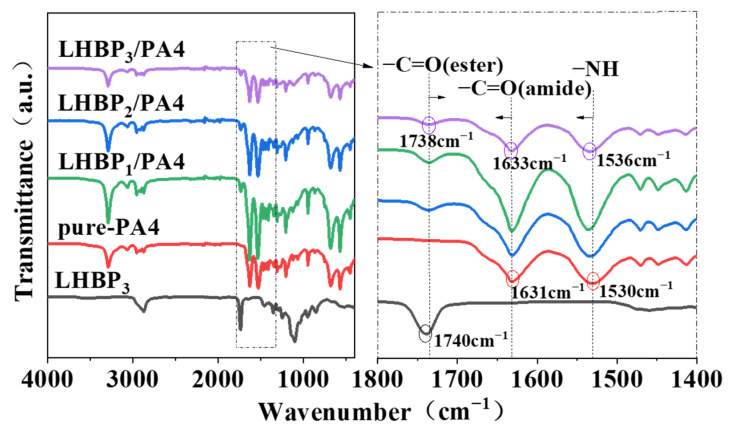
FTIR spectra of LHBP_3_, pure-PA4, and LHBPx/PA4 polymer blends.

The FTIR spectra of pure-PA4 and LHBPx/PA4 polymer blends is shown in Figure 4 [32]. The peaks observed at 1631 and 1530 cm^−1^ correspond to the amide I band (C=O stretching) and the amide II band (N-H bending and C-N stretching) of PA4, respectively. The peak at 1738 cm^−1^ correspond to the carbonyl (C=O stretching) of LHBPx. The main chain of the PA4 molecule contained a significant number of amide groups, and it exhibited intermolecular hydrogen bonds between N-H and C=O. Upon incorporation of LHBPx, a red shift was observed in the C=O stretching vibration peak of PA4 from 1631 cm^−1^ to 1633 cm^−1^ (LHBP_3_/PA4); a similar red shift was also noted in the N-H stretching vibration peak from 1530 cm^−1^ to 1536 cm^−1^ (LHBP_3_/PA4). These observations suggest that hydrogen bond interaction between PA4 molecular chains were weakened by the addition of LHBPx. The carbonyl (C=O) peak on LHBP_3_ shifted from 1740 to 1738 cm^−1^ (LHBP_3_/PA4), indicating an increased polarity of the carbonyl (C=O) group on LHBPx due to the formation of “N-H···O” hydrogen bonds with PA4. The establishment of a hydrogen bond network between LHBPx and PA4 at the molecular level enhanced their compatibility.

### 3.3. Thermal Properties of LHBPx/PA4 Polymer Blend

**Figure 5:**
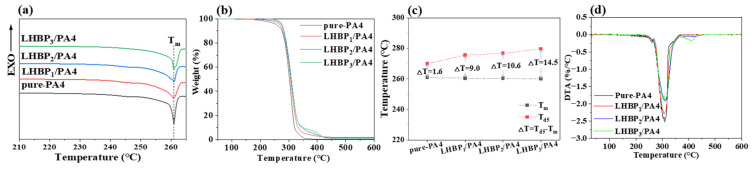
(**a**) DSC curves of pure-PA4 and LHBPx/PA4 polymer blends, (**b**) TGA curves of pure-PA4 and LHBPx/PA4 polymer blends, (**c**) DTG curves for pure-PA4 and LHBPx/PA4 polymer blends, (**d**) T_m_ and T_d5_ changes of pure-PA4 and LHBPx/PA4 polymer blends.

Figure 5a–c shows the DSC, TGA, and DTG curves of pure-PA4 and LHBPx/PA4 polymer blends. Figure 5d shows the changes in T_m_ and T_d5_ of LHBPx/PA4 polymer blends with the different types of LHBPx. With the increase in the branched-chain length of LHBPx, T_m_ gradually decreased from 260.9 °C (pure-PA4) to 260.5 °C (LHBP_1_/PA4), 260.4 °C (LHBP_2_/PA4), and 260.0 °C (LHBP_3_/PA4), and the melting point decreased slightly; T_d5_ gradually increased from 262.5 °C (pure-PA4) to 269.5 °C (LHBP_1_/PA4), 271 °C (LHBP_2_/PA4), and 274.5 °C (LHBP_3_/PA4), and the thermal stability was improved; △T (T_d5_−T_m_) gradually widened from 1.6 °C (pure-PA4) to 9.0 °C (LHBP_1_/PA4), 10.6 °C (LHBP_2_/PA4), and 14.5 °C (LHBP_3_/PA4), and △T widened significantly. With the increase in branch chain length, the △T value increased due to the enhanced thermal stability of LHBPx with longer branches (Figure S2).

As can be seen from Table 4, the addition of LHBP_1_ to the polymerization blends increased the T_d5_ and T_d50_ of LHBP_1_/PA4 by 7 °C and 4 °C, respectively, and that of LHBP_3_/PA4 by 12 °C and 6 °C, respectively, compared to pure-PA4. In contrast, adding LHBPx increased the degradation temperatures of the materials. The degradation temperature of the material increased after mixing with LHBPx. This indicates that the branch length of LHBPx affects the thermal properties of the PA4 matrix, and the longer branch length of LHBPx is more effective in improving the thermal stability of PA4.

Therefore, the highly thermally stable LHBPx formed a physical barrier in PA4, hindering heat conduction, and thereby improving the thermal stability of the LHBPx/PA4 polymer blends.

### 3.4. Rheological Properties of LHBPx/PA4 Polymer Blends

**Figure 6:**
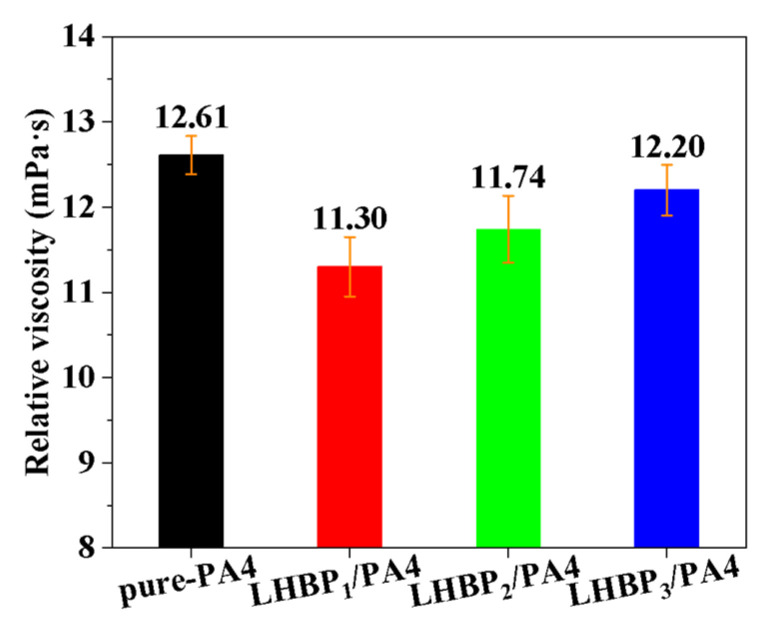
Relative viscosity of pure-PA4 and LHBPx/PA4 polymer blends.

The thermal processability of the polymer is not only related to its thermal properties but is also concerned with its rheological properties. The relative viscosity of the solutions of pure-PA4 and LHBPx/PA4 polymer blends is shown in Figure 6.

The addition of LHBPx reduced the η_r_ of PA4 from 12.61 mPa·s (pure-PA4) to 11.30 mPa·s (LHBP_1_/PA4) at first, and with the increase in LHBPx branched-chain length, η_r_ gradually rose to 11.74 mPa·s (LHBP_2_/PA4) and 12.20 mPa·s (LHBP_3_/PA4). This indicates that a LHBPx with low viscosity could improve the rheology of PA4. The increase in the length of the LHBPx branched chains led to a higher formation of physical entanglements with PA4, thereby enhancing the viscosity of PA4 when a LHBPx with a longer branched chain was added.

### 3.5. Mechanical Properties of LHBPx/PA4 Polymer Blends

**Figure 7:**
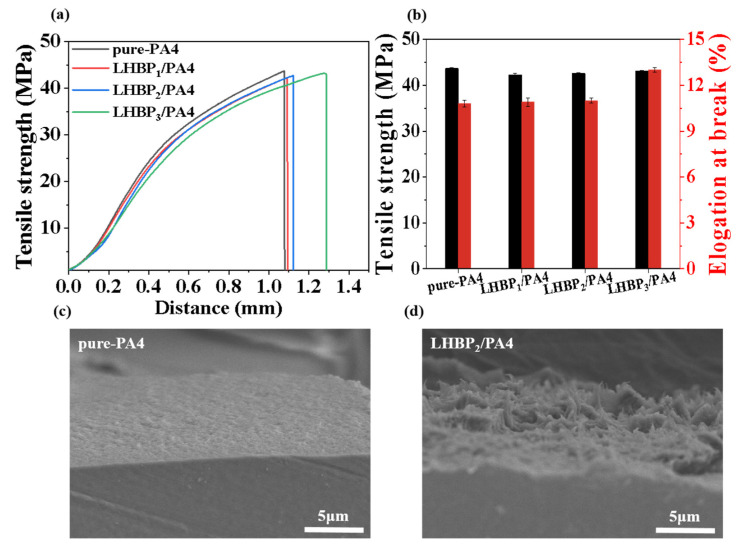
(**a**,**b**) Tensile strength and elongation at break of pure-PA4 and LHBPx/PA4 polymer blend, (**c**) SEM pattern of pure-PA4, (**d**) SEM pattern of LHBP_2_/PA4.

The effect of the type of LHBPx of the LHBPx/PA4 polymer blend on mechanical properties is shown in Figure 7a,b and Table 5. The addition of LHBPx reduced the tensile strength of PA4 from 43.7 MPa (pure-PA4) to 42.3 MPa (LHBP_1_/PA4) at first, and with the increase in LHBPx branched-chain length, tensile strength gradually rose to 42.6 MPa (LHBP_2_/PA4) and 43.1 MPa (LHBP_3_/PA4); elongation at break gradually increased from 10.8% (pure-PA4) to 10.9 (LHBP_1_/PA4), 11.1 (LHBP_2_/PA4), and 13.0 (LHBP_3_/PA4); the elongation at break was improved. Therefore, when an appropriate amount of LHBPx was added, the toughness of PA4 could be improved to a certain extent, and the mechanical strength did not decrease significantly.

The microstructure of the tensile section of pure-PA4 and the LHBPx/PA4 polymer blends was observed by SEM micrographs (Figure 7c,d). The tensile section of pure-PA4 had no obvious deformation, and the fracture was flat and smooth, which was an obvious brittle fracture. With the addition of 10 wt.% LHBPx, the LHBPx/PA4 composite tensile section had obvious plastic deformation, the fracture was rough, and the fiber area was visible, which was due to the occurrence of holes in the section, and the hole-induced ductile fracture made the section rough. The SEM test further proved that with the addition of LHBPx, the mode of tensile fracture transitions changed from brittle to ductile. These results indicate that LHBPx could obviously improve the toughness of PA4.

### 3.6. Crystallization Behavior of LHBPx/PA4 Polymer Blend

**Figure 8:**
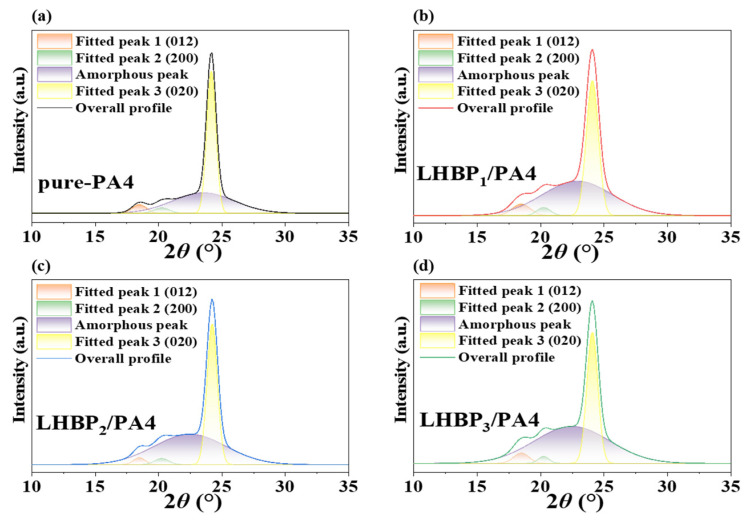
XRD spectra of pure-PA4 and LHBPx/PA4 polymer blends, (**a**) pure-PA4, (**b**) LHBP_1_/PA4, (**c**) LHBP_2_/PA4, (**d**) LHBP_3_/PA4.

The properties of PA4 are mainly determined by the crystallization behavior, which can control the formation of the microstructure. The crystallization parameters of pure PA4 and LHBPx/PA4 polymer blends were analyzed by XRD, as shown in Figure 8, and the fitting results are shown in Table 6. The crystallinity data of LHBPx/PA4 samples were calculated by substituting the fitted data into Equation (1) [33,34].

(1) $$X_{Crystallinity}=\frac{\left[A_{\left(012\right)}+A_{\left(200\right)}+A_{\left(020\right)}\right]}{\left[A_{\left(012\right)}+A_{\left(200\right)}+A_{\left(020\right)}+A_{\left(mes-form\right)}\right]}$$

LHBPx/PA4 polymer blends were fitted with three crystal-plane diffraction peaks and one amorphous peak. The diffraction peaks of the three crystal planes were located at 2θ ≈ 18.5°, 2θ ≈ 20.3°, and 2θ ≈ 24.1°, respectively, corresponding to the (012) crystal plane, (200) crystal plane, and (020) crystal plane of the PA4. The (200) crystal plane and (020) crystal plane belonged to the α crystal form of PA4. The α crystal form was a relatively stable structure formed by hydrogen bonds between antiparallel molecular chains [35,36,37]. The (200) crystal faces were connected by hydrogen bonds, and the spacing of the crystal faces was related to the strength of the PA4 hydrogen bond [33]. With the increase in the branched-chain length of LHBPx, the (012) and (200) crystal plane diffraction peak area ratio A_1_/A_2_ gradually decreased, indicating that increasing the branched-chain length of LHBPx promoted the growth of the (200) crystal planes and inhibited the growth of the (012) crystal planes. The crystallinity of LHBPx/PA4 also decreased as the branched-chain length of LHBPx increased. The addition of LHBPx reduced the crystallinity, but promoted the growth of a stable α crystal form of PA4 [38,39], so the melting point of the composite did not drop significantly.

### 3.7. Non-Isothermal Crystallization Kinetic Analysis of LHBPx/PA4 Polymer Blends

**Figure 9:**
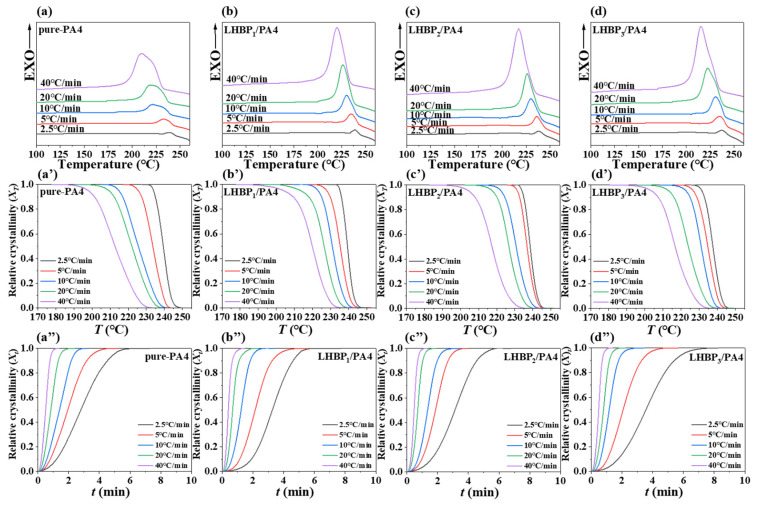
(**a**–**d**) DSC curves of pure-PA4 and LHBPx/PA4 polymer blends at different cooling rates, (**a**’–**d**’) relationship between relative crystallinity *X_T_* and crystallization temperature T of pure-PA4 and LHBPx/PA4 polymer blends, (**a**”–**d**”) relationship between relative crystallinity *X_t_* and crystallization temperature t of pure-PA4 and LHBPx/PA4 polymer blends.

To understand the crystallization process of the LHBPx/PA4 polymer blends, they were crystallized at five different cooling rates (2.5, 5, 10, 20, 40 °C·min^−1^, respectively). The DSC curves of pure-PA4 and LHBPx/PA4 polymer blends under various cooling rates are shown in Figure 9a–d.

At different crystallization rates, the crystallization peaks of the polymer blends exhibit unimodality, which is the crystallization temperature attributed to the α crystal type of PA4 [11]. With an increased cooling rate, the exothermic peak of the crystal broadened and shifted toward lower temperatures. Similarly, the initial, peak and final temperatures at the crystallization of all polymer blends also shifted toward lower temperatures. The observed phenomenon could be attributed to a delay in the movement of PA4 molecular chains related to the cooling rate. Consequently, these molecular chains were unable to sufficiently integrate into the lattice structure and become immobilized, resulting in imperfect crystallization. Conversely, when the cooling rate slowed, there was ample time for molecular chain adjustment within the lattice structure, leading to relatively flawless crystal formation. As a result, narrower crystallization peaks were observed with higher initiation and peak temperatures. For the LHBPx/PA4 blend system, as the length of the LHBPx branched chain decreased, the crystallization peak of the PA4 phases became sharper and moved toward the high-temperature direction, indicating that the LHBPx/PA4 polymer blends with a shorter branched chain of LHBP were more easily crystallized.

The relative crystallinity $X\left(T\right)$ of the blends at a specified temperature can be calculated using Equation (2) [40]:

(2) $$X\left(T\right)=\frac{Q_{T}}{Q_{T_{\infty }}}=\frac{\int_{T_{0}}^{T}\left(\mathrm{d}H/\mathrm{d}T\right)\mathrm{d}T}{\int_{T_{0}}^{T_{\infty }}\left(\mathrm{d}H/dT\right)\mathrm{d}T}$$

where $T_{0}$ is the temperature at the beginning of crystallization, $T_{\infty }$ is the temperature when the crystallization is complete, $Q_{T}$ and $Q_{T_{\infty }}$ are the heat released when the crystallinity is $T_{0}$ and the crystallinity is $T_{\infty }$, and ⅆH/ⅆT is the heat flow at temperature T.

The relationship between crystallization time (t) and crystallization temperature (T) is shown in Equation (3) [40], as follows:

(3) $$t=\frac{\left|T_{0}-T\right|}{\Phi }\;$$

where Φ is the cooling rate. By bringing Equation (3) into Equation (2), the relative crystallinity $X\left(t\right)$ with respect to crystallization time (t) is obtained.

The $X\left(T\right)-T$ and $X\left(t\right)-t$ diagrams of LHBP/PA4 polymer blends obtained using Equations (2) and (3) are shown in Figure 9a’–d’ and Figure 9a”–d”, respectively.

With the increase in cooling rate, the $X\left(T\right)-T$ curves of the four samples all moved towards lower temperatures. At 234 °C, the $X\left(T\right)$ of pure-PA4 was 100% when cooled at a rate of 2.5 °C·min^−1^, whereas it dropped to 0% when cooled at a rate of 20 °C·min^−1^. This clearly demonstrated that the cooling rate significantly influenced the crystallization process of PA4.

The $X\left(t\right)-t$ curves of the four samples were all ‘S’ curves, which were because the crystal growth mainly went through three stages: crystal nucleus growth, crystal growth, and crystal stability. With the increase in cooling rate, the slope of the crystal growth stage gradually increased, indicating that the crystallization rate of the polymer increased with the increase in cooling rate. The slope of PA4 added with LHBPx became larger in the crystal growth stage, that is, at the same cooling rate, the time required for $X\left(t\right)$ = 50% was shorter. The addition of LHBPx led to an increased number of heterophase points and promoted the formation of crystal nuclei in PA4, thereby enhancing the crystallization rate during the crystal nucleus growth stage. However, the entanglement of long branched-chain LHBP hindered the further arrangement of PA4 molecular chains and, consequently, reduced the crystallinity of PA4.

**Figure 10:**
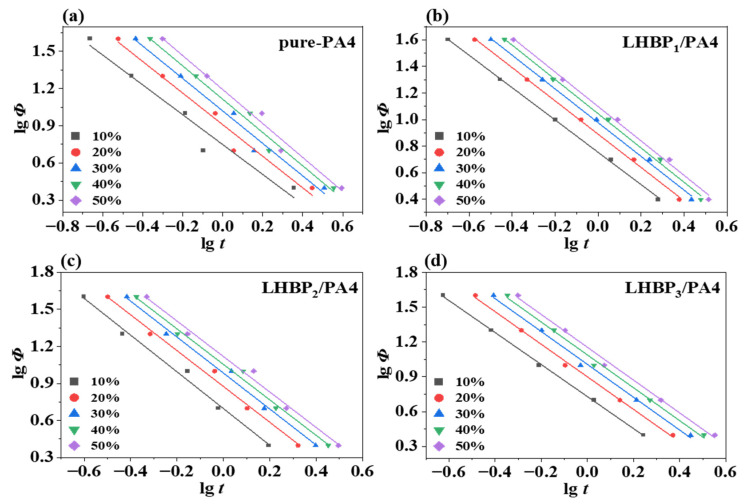
lg⁡Φ−lg⁡t curves of Mo’s method, (**a**) pure-PA4, (**b**) LHBP_1_/PA4, (**c**) LHBP_2_/PA4, (**d**) LHBP_3_/PA4.

Mo’s (Equation (6)) [41,42] method was combined with the Avrami (Equation (4)) [41] and Ozawa (Equation (5)) [43] methods to obtain a wider range of non-isothermal crystallization kinetics methods. So, the crystallization kinetic parameters of the polymer blend were fitted and calculated using Mo’s method, and the results are shown in Figure 10 and Table 7, as follows:

(4) $$\mathrm{lg}\left[-\ln\left(1-X\left(t\right)\right)\right]=\mathrm{lg}Zc+n\mathrm{lg}t$$

where n is the Avrami index, which is related to the nucleation mechanism and crystal growth dimension, Zc is the crystallization rate constant, $X\left(t\right)$ is the relative crystallinity of different times, and t is the time, and the following:

(5) $$\mathrm{lg}\left[-\ln\left(1-X\left(T\right)\right)\right]=\mathrm{lg}K\left(T\right)-m\mathrm{lg}\Phi$$

where $K\left(T\right)$ is the function of temperature, Φ is the cooling rate, m is the Ozawa index, and $X\left(T\right)$ is the relative crystallinity of different temperatures, and the following:

(6) $$\mathrm{lg}\Phi =\mathrm{lg}F\left(T\right)-a\cdot \mathrm{lg}t$$

where $F\left(T\right)$ is the cooling rate required to reach a certain relative crystallinity per unit of time; a=n/m, n, and m are the Avrami and Ozawa indexes for the non-isothermal crystallization process, respectively.

The $F\left(T\right)$ value can represent the crystallization rate. Under the same relative crystallinity, the higher the value, the slower the crystallization rate. When the LHBP of the shorter-chain branch was added, $F\left(T\right)$ became smaller. With the gradual increase in the length of the branched chain, $F\left(T\right)$ became bigger. The results indicated that the crystallization rate of PA4 was first accelerated and then slowed down with the increase in LHBP branched chain length; a=n/m, from which the Ozawa index, m could be calculated (Table 7). This result proved that pure-PA4 grew in two dimensions, while PA4 with LHBPx added grew in three dimensions, and both homogeneous nucleation and heterophase nucleation occurred in the crystallization process of the four materials.

The Kissinger (Equation (7)) [43] method was used to calculate the non-isothermal crystallization activation energy of the polymer blends, as follows:

(7) $$\ln\left(\frac{\Phi }{T_{c}^{2}}\right)=\ln\left(\frac{A\cdot R}{\Delta E}\right)-\frac{\Delta E}{R\cdot T_{c}}$$

where Φ is the cooling rate, $T_{c}$ is the temperature corresponding to the cooling crystallization peak, A is the frequency factor independent of the cooling rate, R is the ideal gas constant (R = 8.314 J·(K·mol)^−1^), and ΔE is the crystallization activation energy.

**Figure 11:**
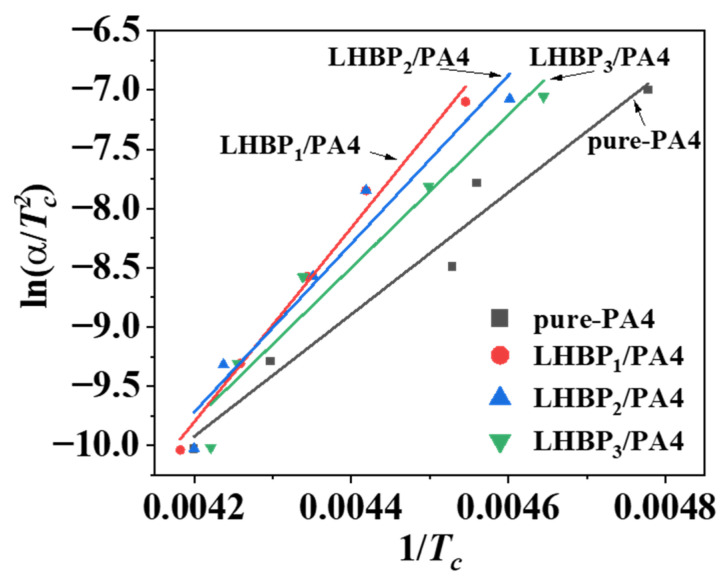
*ln*($a/T_{c}^{2}$) − 1/$T_{c}$ curves of Kissinger equation.

Using *ln*($\Phi /T_{c}^{2}$) to plot 1/$T_{c}$, a linear correlation line could be obtained (Figure 11), which could be used to calculate ΔE from the slope of the line. Therefore, ΔE_0_ (pure-PA4) = −42.82 KJ·mol^−1^, ΔE_1_ (LHBP_1_/PA4) = −68.14 KJ·mol^−1^, ΔE_2_ (LHBP_2_/PA4) = −59.07 KJ·mol^−1^, and ΔE_3_ (LHBP_3_/PA4) = −53.65 KJ·mol^−1^. The value of crystallization activation energy was negative, indicating that the process could be a spontaneous exothermic process. The crystallization activation energy of PA4 with LHBPx added was lower, which further indicates that PA4 with LHBP added was more prone to crystallization. However, since crystal growth included the nucleation and growth processes, LHBPx promoted the nucleation of PA4, but hindered the growth process of PA4, resulting in an increase in crystallization rate but a decrease in crystallinity. With the increase in LHBPx branched-chain length, the crystallization activation energy of the LHBPx/PA4 polymer blends gradually increased and became more difficult to crystallize. The reason for this was that when the additional amount was the same, hyperbranched polymers with longer branched chains had a higher relative molecular weight.

## 4. Conclusions

In this study, the incorporation of LHBPx enhanced the thermal processability of PA4, improving its toughness while maintaining mechanical strength. Moreover, the longer the branched chain of LHBPx was, the more pronounced the improvement in the thermal processability and toughness of PA4 became. Compared with PA4, the thermal processability window was enhanced to 14.5 °C (LHBP_3_/PA4), and the rheological effect was increased; thereby, the thermal processability of LHBPx/PA4 was improved. The elongation at the break of the LHBP_3_/PA4 polymer blend was increased by 20.4% compared with PA4, and the brittle fracture was changed into a ductile fracture. The crystallinity of PA4, when blended with LHBPx, was reduced from 54.41% to 37.42%, but the crystal type did not change significantly, so the T_m_ of PA4 almost had no change. In summary, this study provided a research idea to modify the thermal processability and toughness of PA4, which was meaningful in promoting the further development of PA4 in industrial plastics.

## Figures and Tables

**Table 1:** Types and amounts of A_2_-X and B_4_ required for LHBPx synthesis.

Sample	A_2_-1 (mL)	A_2_-2 (mL)	A_2_-3 (mL)	B_4_ (mL)
LHBP_1_	4.2	/	/	5.8
LHBP_2_	/	5.9	/	4.1
LHBP_3_	/	/	7.8	2.2

**Table 2:** Compositions of the LHBPx/PA4 polymer blends.

Sample	LHBP_1_ (wt.%)	LHBP_2_ (wt.%)	LHBP_3_ (wt.%)	PA4 (wt.%)
LHBP_1_/PA4	10	/	/	90
LHBP_2_/PA4	/	10	/	90
LHBP_3_/PA4	/		10	90

**Table 3:** Mw, Mn, and PDI of LHBPx.

Sample	M_w_	M_n_	PDI
LHBP_1_	15,305	3303	4.6
LHBP_2_	24,660	4244	5.8
LHBP_3_	17,502	5502	3.2

**Table 4:** T_d5_, T_d50_, and DTG peaks of pure-PA4 and LHBPx/PA4 polymer blends.

Sample	T_d5_ (°C)	T_d50_ (°C)	DTG Peak 1	
			T_deg1_ (°C)	TG (%)
pure-PA4	262.5	302.0	308.0	36.0
LHBP_1_/PA4	269.5	306.0	310.0	42.4
LHBP_2_/PA4	271.0	308.0	311.0	44.0
LHBP_3_/PA4	274.5	308.0	312.0	43.9

**Table 5:** Tensile properties data sheet for pure-PA4 and LHBPx/PA4 samples.

Sample	Tensile Strength (MPa)	Elongation at Break (%)	Young’s Modulus (MPa)
pure-PA4	43.7	10.8	404.6
LHBP_1_/PA4	42.3	10.9	388.1
LHBP_2_/PA4	42.6	11.0	387.3
LHBP_3_/PA4	43.1	13.0	331.5

**Table 6:** Peak-fitting area proportion and crystallinity of pure-PA4 and LHBPx/PA4 polymer blends.

Sample	A_1_ (%) (012)	A_2_ (%) (200)	A_1_/A_2_	Crystallinity (%)
pure-PA4	4.35	2.98	1.46	54.41
LHBP_1_/PA4	3.61	2.96	1.22	47.62
LHBP_2_/PA4	3.06	2.70	1.13	43.96
LHBP_3_/PA4	2.44	2.44	1.00	37.42

**Table 7:** Mo’s method data for pure-PA4 and LHBPx/PA4 polymer blends.

Sample	$X_{t}$(%)	*F*(*T*)	*R* ^2^	*a*	*m*
pure-PA4	10	14.27	0.89	1.17	1.99–2.04
	20	16.45	0.93	1.18	
	30	18.82	0.95	1.20	
	40	20.69	0.97	1.19	
	50	23.03	0.97	1.20	
LHBP_1_/PA4	10	10.57	0.97	0.97	2.94–3.06
	20	12.09	0.98	0.99	
	30	13.21	0.98	1.00	
	40	13.96	0.98	1.00	
	50	14.96	0.98	1.01	
LHBP_2_/PA4	10	12.29	0.92	1.04	2.60–2.73
	20	14.02	0.95	1.06	
	30	15.15	0.97	1.06	
	40	16.28	0.97	1.08	
	50	17.52	0.98	1.09	
LHBP_3_/PA4	10	17.04	0.96	1.28	2.08–2.20
	20	19.65	0.97	1.25	
	30	21.61	0.98	1.22	
	40	23.35	0.98	1.21	
	50	24.97	0.98	1.22	

## Data Availability

The original contributions presented in this study are included in the article/supplementary material. Further inquiries can be directed to the corresponding author.

## Supplementary Materials

The following supporting information can be downloaded at: https://www.mdpi.com/article/10.3390/polym17030318/s1, Figure S1: ^1^H-NMR spectra of A_2_-1, B_4_, TMC and LHBP_1_; Figure S2: TGA pattern of LHBPx.
