# Supplementary material for: Thermal, Crystallization, and Toughness Behavior of Polyamide 4/Long-Chain Hyperbranched Polymer Blends
Source: Polymers (Basel). 2025 Jan 24;17(3):318. doi: 10.3390/polym17030318 (PMC11820697; doi:10.3390/polym17030318)
Supplement: Supplementary file 1 [file polymers-17-00318-s001.zip › polymers-3427424-supplementary.pdf]

Supporting Information

Title: The effect of long-chain hyperbranched polymer on the thermal and toughness properties of polyamide 4

Dr. Xiaomin Zhao

College of materials science and engineering in Huaqiao University

Email: xiaominelena@163.com

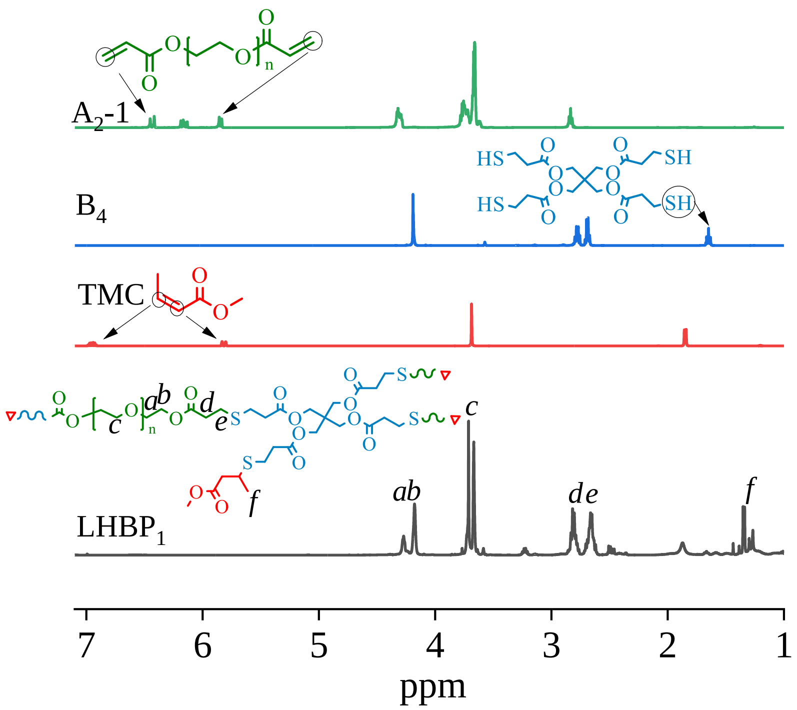

Figure S1: ^{1}H-NMR spectra of A_2-1, B_4, TMC and $LHBP_1$

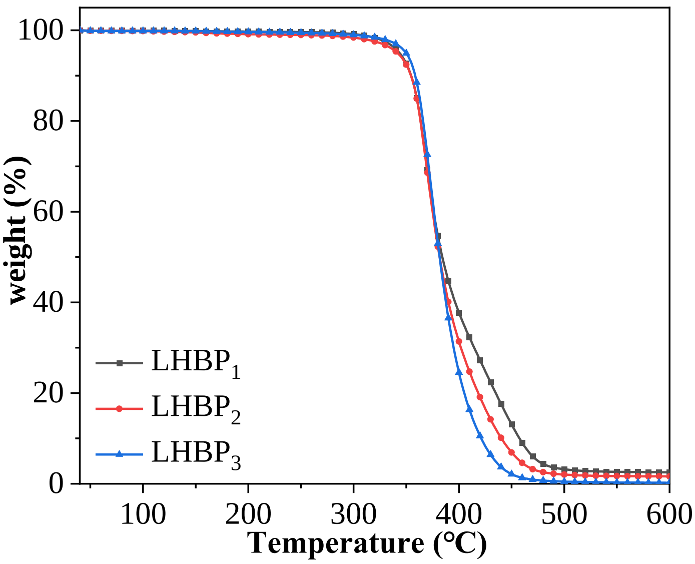

Figure S2: TGA pattern of LHBPx
